# Integrative and complementary practices in health, nurses’ profile and care provided to people with hypertension: a mixed study design *

**DOI:** 10.1590/1518-8345.6287.3915

**Published:** 2023-05-15

**Authors:** Daiana Cristina Wickert, Daniela Dallegrave, Diéssica Roggia Piexak, Marlise Capa Verde Almeida de Mello, Laís Mara Caetano da Silva Corcini, Maria Denise Schimith

**Affiliations:** 1 Universidade Federal de Santa Maria, Santa Maria, RS, Brasil.; 2 Becaria de la Coordenação de Aperfeiçoamento de Pessoal de Nível Superior (CAPES), Brasil.; 3 Universidade Federal do Rio Grande do Sul, Departamento de Assistência e Orientação Profissional da Escola de Enfermagem, Porto Alegre, RS, Brasil.; 4 Universidade Federal do Rio Grande, Rio Grande, RS, Brasil.; 5 Universidade Federal do Rio Grande, Escola de Enfermagem, Rio Grande, RS, Brasil.

**Keywords:** Nursing, Nursing Care, Complementary Therapies, Traditional Medicine, Integrative Medicine, Hypertension, Enfermería, Atención de Enfermería, Terapias Complementarias, Medicina Tradicional, Medicina Integral, Hipertensión, Enfermagem, Cuidados de Enfermagem, Terapias Complementares, Medicina Tradicional, Medicina Integrativa, Hipertensão

## Abstract

**Objective:**

to analyze the profile of nurses regarding integrative and complementary practices in health (ICPH) and understand how they are used in the care of people with arterial hypertension.

**Method:**

mixed-methods sequential explanatory design. The cross-sectional quantitative stage included 386 nurses who completed an online questionnaire addressing sociodemographic and professional information, training, and practice, with a descriptive and inferential analysis. The qualitative stage was performed via 18 online interviews with professionals who had ICPH training and implemented it in the care provided to individuals with hypertension, with a participatory analysis. Integration occurred through a connecting approach.

**Results:**

36.8% had ICPH training; most were women, Caucasian, married, public servants, aged 37 (+ 9.4) on average; 14.2% incorporated ICPH into the care provided to people with hypertension; predominantly auriculotherapy (28.2%) and bloodletting in hypertensive crises. The results show that nurses integrally approached patients, and their approach was not limited to the vital sign altered at the time, but they also intervened in anxiety, stress, sleep, and rest. A potentiality observed concerns support treatment adherence.

**Conclusion:**

the profile of nurses with ICPH training is presented, and such practice has implications for lowering blood pressure. ICPH has been incorporated into the care of people with hypertension, but its use is still incipient, considering its potential in nursing care.

## Abstract (es)

**Objetivo:**

analizar el perfil de enfermeras(os) acerca de las prácticas integradoras y complementarias en salud y comprender cómo son utilizadas en el cuidado a personas con hipertensión arterial.

**Método:**

explicativo, secuencial y mixto. La etapa cuantitativa transversal contó con 386 enfermeras(os), a través de cuestionario virtual, abordando el perfil sociodemográfico y profesional y la formación y actuación, con análisis descriptivo e inferencial. La etapa cualitativa se realizó mediante 18 entrevistas virtuales con profesionales que poseían formación en las prácticas y las utilizaban en el cuidado a personas con hipertensión, fundamentadas en el análisis participativo. La integración se realizó por conexión.

**Resultados:**

el 36,8% tuvo formación en las prácticas con predominio de mujeres, blancas, casadas, funcionarias públicas, con media de edad de 37 años (+ 9,4). El 14,2% utilizaba las prácticas en el cuidado en personas con hipertensión, predominando la auriculoterapia (28,2%) y la sangría en la crisis hipertensiva. Se evidenció el abordaje integral del paciente, no limitado a signos vitales alterados, con intervención en la ansiedad, estrés, sueño y reposo. Como potencialidad, tenemos el auxilio en la adhesión al tratamiento.

**Conclusión:**

se presentó el perfil de enfermeras(os) con formación en prácticas integradoras y complementarias. Se comprende que esas prácticas tienen implicación en la disminución de la presión arterial y que son utilizadas en el cuidado a personas con hipertensión, sin embargo de manera incipiente, considerando el potencial en el cuidado de enfermería.

## Abstract (pt)

**Objetivo:**

analisar o perfil de enfermeiras(os) acerca das práticas integrativas e complementares em saúde e compreender como são utilizadas no cuidado às pessoas com hipertensão arterial.

**Método:**

explanatório sequencial misto. A etapa quantitativa transversal contou com 386 enfermeiras(os), via questionário virtual, abordando perfil sociodemográfico e profissional, formação e atuação, com análise descritiva e inferencial. A etapa qualitativa ocorreu mediante 18 entrevistas virtuais com profissionais que possuem formação nas práticas e as utilizam no cuidado às pessoas com hipertensão, fundamentada na análise participativa. A integração se deu por conexão.

**Resultados:**

36,8% tinham formação nas práticas, predominando mulheres, brancas, casadas, servidoras públicas, com média de idade de 37 anos (+ 9,4). 14,2% utilizam as práticas no cuidado às pessoas com hipertensão, predominando a auriculoterapia (28,2%) e a sangria na crise hipertensiva. Evidenciou-se a abordagem integral do paciente, não limitada ao sinal vital alterado, com intervenção na ansiedade, estresse, sono e repouso. Como potencialidade, tem-se o auxílio na adesão ao tratamento.

**Conclusão:**

apresentou-se o perfil de enfermeiras(os) com formação em práticas integrativas e complementares. Compreende-se que tais práticas têm implicação na diminuição da pressão arterial, e são utilizadas no cuidado às pessoas com hipertensão, porém de forma incipiente, considerando o potencial no cuidado de enfermagem.

**Highlights:**

**(1)** Presents the profile of nurses with ICPH training in Santa Catarina, Brazil

**(2)** Evidence of ICPH and how it is incorporated into the care provided to people with hypertension.

**(3)** Potentialities regarding incorporating ICPH into the care provided to people with hypertension.

**(4)** Challenges faced when incorporating ICPH into the care provided to people with hypertension.

**(5)** Strengths and evidence of the nurses’ protagonism in the adoption of ICPH.

## Introduction

Non-communicable diseases (NCDs) are a health problem worldwide, accounting for approximately 41 million deaths every year; it represents 74% of deaths in Brazil ^( 1)^ . The global and Brazilian targets for decreasing NCDs include the prevention of diseases and health promotion based on reducing risk factors, such as arterial hypertension (AH), characterized by persistently elevated blood pressure (BP) (systolic greater than or equal to 140 mmHg and/or diastolic greater than or equal to 90 mmHg) ^( 2)^ . 

Integrative and Complementary Practices in Health (ICPH) have been used throughout history to maintain health and prevent and treat diseases, particularly chronic diseases ^( 3)^ . Scientific evidence shows that ICPH is incorporated into the care of individuals with AH worldwide ^( 4)^ . However, a recent review of theses and dissertations performed in Brazil shows that 11 manuscripts addressed ICPH in the context of AH management, two were conducted by nurses, and one focused on the nurses’ practice, highlighting an important gap in the literature ^( 5)^ . 

In addition to pharmacological interventions, the precepts of integrative nursing, a field on the rise, consider the concomitant use of non-pharmacological interventions scientifically proven to be safe and effective ^( 6)^ , such as ICPH. The nursing field was the first to recognize complementary therapies as a professional practice in 1997 ^( 7)^ . Currently, the Resolution of the Federal Nursing Council (COFEN) No, 625/2020 updated and ensured the support of Nursing specialties in ICPH, including phytotherapy, homeopathy, orthomolecular therapy, floral therapy, foot reflexology, *Reiki*, yoga, therapeutic touch, music therapy, color therapy, hypnosis, and acupuncture ^( 8)^ . 

Nevertheless, the profile of professionals working with ICPH is still unknown in Brazil, though there is an emphasis on nurses implementing it besides research and extension. ICPH can expand the practice of nurses, providing greater autonomy and improving the quality of the care provided ^( 9)^ . 

In line with the strategy of the World Health Organization (WHO) on traditional medicine (2014-2023) ^( 3)^ , it is worth noting a scarcity of guidelines regulating training programs in the Brazilian context. Additionally, the profile of nurses working with ICPH is unknown, as well as the concepts of this practice and how it is implemented in the nursing care provided to individuals with AH, which is the research problem addressed here. 

Filling in such gaps will strengthen the ICPH in the Brazilian Unified Health System (SUS), the ICPH training of nurses, and the care nurses provide to individuals with AH, which justifies this investigation. Thus, this study’s objective was to analyze the profile of nurses regarding Traditional, Complementary, and Integrative Medicine and understand how it is incorporated into the care provided to individuals with high blood pressure.

## Method

### Study design

A mixed-methods sequential explanatory design was adopted in this study, in which quantitative data (QUAN) were collected and analyzed first (with greater weight). The qualitative stage (QUAL) was based on the results obtained in the quantitative stage ^( 10)^ , and integration occurred through a connecting approach. Mixed methods were used to deepen the understanding of the research problem, with the quantitative stage having a cross-sectional design and the qualitative stage based on participatory analysis ^( 11)^ . The Strengthening the Reporting of Observational Studies in Epidemiology (STROBE) and Standards for Reporting Qualitative Research (SRQR) were adopted. 

### Study setting, population, and sample

As part of a national multicenter project, the study setting was Santa Catarina (SC), Brazil, and the population consisted of the nurses working in that state. There were 16,620 nurses registered with the Regional Nursing Council of SC ^( 12)^ when the project was submitted to the Institutional Review Board. A simple random sampling calculation for finite populations yielded a minimum sample of 376 professionals. The inclusion criteria for the quantitative stage were having an undergraduate degree in nursing and an occupation in SC. 

Considering our interest in understanding the adoption of ICPH to manage AH, the participants for the qualitative stage were drawn among those who participated in the quantitative stage and reported the use of ICPH in the care provided to people with AH. Thus, software freely available online was used to randomly select the participants and ensure representativeness. At least two nurses per health macro-region were selected; the number of participants varied according to data saturation. Santa Catarina has seven health macro-regions: 1-Grande Oeste, 2-Meio Oeste e Serra Catarinense, 3-Planalto Norte and Nordeste, 4-Foz do Rio Itajaí, 5-Vale do Itajaí, 6-Grande Florianópolis, and 7-Sul. Three nurses from macro-regions 1, 5, and 7 refused to participate due to a lack of time; one nurse from macro-region 1 refused for not feeling apt due to a lack of experience with ICPH; and one from macro-region 3 refused because she worked in urgency and emergency and did not implement ICPH. These macro-regions were included in new draws following the geographically closest regions. Macro-region 4 did not have nurses who met the inclusion criteria; macro-region 2 had only one nurse. Macro-region 5 had three nurses who met the inclusion criteria, though only one was available to participate.

### Data collection

Quantitative data were collected online using a 63-item questionnaire, 26 of which were answered by all the nurses. Thirteen questions addressed sociodemographic information, eight concerned the participants’ professional profiles, and five addressed their qualifications. The remaining 36 questions were specific to nurses who attended a training program on ICPH (18 questions concerned ICPH training, and 18 addressed professional practice). The final question asked whether they would be available for an online interview (qualitative stage). Consent was manifested by reading the free and informed consent form and checking the box “I agree to participate in the study.”

The questionnaire was developed in the LimeSurvey software and was peer-reviewed by four nurses from the south qualified in ICPH and/or mixed methods. The objective was to adapt the questions’ language and organization. Next, a pilot study was implemented with six participants from five Brazilian regions.

The survey was publicized via WhatsApp, Facebook, Instagram, institutional websites, and e-mail. Due to the difficulties imposed by the COVID-19 pandemic, we collected contacts freely accessible on universities’ websites and used the pyramid strategy, in which each new participant nominated new participants. In addition, volunteers and scholarship holders were trained via online meetings to assist in disseminating the study and data collection from June 16 ^th^, 2021, to October 15 ^th^, 2021, reaching a sample of 386 participants. 

The online interviews were initiated after identifying the nurses who incorporated ICPH in the care delivered to individuals with AH. A semi-structured script containing 12 questions addressing training, practice, potentialities, and challenges faced in the care provided to people with HA was used; a pilot test was performed with a nurse from Rio Grande do Sul to qualify the script.

Using the Google Meet Platform, the first author held the online interviews individually (to minimize dissonance in the data collection process) from January 11 ^th^, 2021, to December 20 ^th^, 2021. The video calls were made using an institutional e-mail to protect data and lasted 47 minutes on average. The consent form had been previously sent via e-mail, which was read and video recorded before initiating the interviews; the participants also agreed with the interviews being video recorded. The framework adopted for analysis demanded that a consensus be reached on the narrative that resulted from each interview; hence, two video calls were needed, one for the interview and one for its validation. The latter lasted 31 minutes on average. 

### Data treatment and analysis

Quantitative data were analyzed descriptively and inferentially using the Statistical Product and Service Solutions (SPSS) ^®^ 26.0 and Epi Info™ 7.2 softwares (CDC, Atlanta, USA). First, a descriptive analysis was performed considering the sample’s characteristics. After calculating the prevalence of ICPH training and associated factors, the relationship between the variables was assessed using the Chi-square test, with significance established at a p-value <0.05 in the two-tailed test. The Fisher’s Exact Test was adopted for small samples (below five in each subcategory). 

The measure of association adopted was the prevalence ratio (PR), with its respective 95% confidence intervals. In addition, the hypothesis of a linear relationship between the two variables was investigated for variables with more than two categories using the Mantel-Haenszel Chi-Square. When the Breslow-Day Test for the ratio interaction showed a value >0.05, the PR was adjusted for the variable under analysis.

The interviews were recorded and manually transcribed in Microsoft Word. The 18 participants were identified by the names of crystals, followed by their ages, higher academic training, and health macro-region to which they belonged.

Participatory analysis of the interviews occurred at three points: 1 ^st^ (narrative construction), in which the interviews were recorded and transcribed verbatim. The transcriptions originated the narratives, which were previously analyzed; 2 ^nd^ (hermeneutics or validation): the narratives were presented to the participants via a video call (Google Meet) to validate data and produce intervention effects, more deeply discussing issues or subjects not much elaborated on in the first discussion; 3 ^rd^ (building consensus): discussion, review of opinions, agreements, and disagreements. After consensus, the narrative was ready for analysis and identifying meaning cores ^( 11)^ . 

After analyzing the quantitative and qualitative data, we proceeded to interpret the mixed methods to integrate the findings according to a connecting approach; data from the quantitative stage were connected to determine the questions and the participants for the qualitative stage. Joint displays show this integration to facilitate visualizing the findings ^( 13)^ . 

### Ethical aspects

The study project was approved by the Institutional Review Board at the hosting (No. 4,618,324) and collaborating centers (No. 4,646,717); all the study’s stages complied with Resolutions No. 466/2012 and No. 510/2016, National Council of Health.

## Results

A total of 386 (100.0%) nurses participated in the quantitative stage; 142 (36.8%) of these had ICPH training, and 55 (14.2%) mentioned they incorporated ICPH in the care provided to people with AH. The socio-demographic profile is presented in Table 1. On average, the participants were 37 years (+ 9.4), and most were between 31 and 35. 

**Table 1 -:** Profile of nurses with and without training in Traditional, Complementary, and Integrative Medicine (ICPH). Santa Catarina, Brazil, 2021 (N=386)

Nurses n (%)	Nurses w/ ICPH* training n (%)
**Gender**		
Female	357 (92.5)	132 (93.0)
Male	29 (7.5)	10 (7.0)
**Marital status**		
Married	146 (37.8)	58 (40.3)
Divorced/Separated	17 (4.4)	6 (4.2)
Single	100 (25.9)	35 (24.3)
Stable union/cohabitate/dating	119 (30.8)	43 (29.9)
Widowed	4 (1.0)	2 (1.4)
**Ethnicity**		
Asian-descendant	2 (0.5)	1 (0.7)
Caucasian	346 (89.6)	130 (91.5)
Mixed race	31 (8.0)	11 (7.7)
Afro-descendant	4 (1.0)	0
None of the above	3 (0.8)	0
**Current employment situation**		
Retired/Pensioner	11 (2.8)	5 (3.5)
Formally employed	93 (24.1)	19 (13.4)
Informally employed	18 (4.7)	5 (3.5)
Self-employed/Social Security	7 (1.8)	3 (2.1)
Self-employed/No Social Security	10 (2.6)	6 (4.2)
Unemployed	7 (1.8)	2 (1.4)
Employer	6 (1.6)	2 (1.4)
Service Provider	31 (8.0)	16 (11.3)
Public servant	225 (58.3)	92 (64.8)
**Legal nature of work**		
Public Health Institution	263 (68.1)	103 (72.5)
Private Health Institution	58 (15.0)	14 (9.9)
Autonomous Health Institution	8 (2.1)	5 (3.5)
Philanthropic Health Institution	27 (7.0)	7 (4.9)
Does not work in nursing	20 (5.2)	6 (4.2)
**Weekly workload**		
20 hours	11 (2.8)	4 (2.8)
36 hours	30 (7.8)	10 (7.0)
40 hours	229 (59.3)	97 (68.3)
44 hours	47 (12.2)	8 (5.6)
More than 44 hours	47 (12.2)	16 (11.3)
Not currently working	14 (3.6)	6 (4.2)
Work by shifts	13 (3.4)	3 (2.1)
**Individual income (M.W. ^†^ R$ 1,035.00)**		
Up to 2 times the M.W.	36 (9.3)	9 (6.3)
From 3 to 4 times the M.W.	163 (42.2)	59 (41.5)
From 5 to 6 times the M.W.	120 (31.3)	53 (35.9)
From 7 to 8 times the M.W.	40 (10.4)	15 (10.6)
More than 9 times the M.W.	27 (7.0)	8 (5.6)

*ICPH = Integrative Complementary Practices in Health; ^†^M.W. = Minimum wage.

Regarding age groups, compared to participants aged up to 30, older professionals presented a more significant prevalence of the outcome; that is, the probability of an older nurse not seeking training in ICPH was 17% higher than a younger nurse not seeking ICPH training. Regarding the time since graduation, 41.0% had graduated more than 60 months ago Table 2. 

**Table 2 -:** Raw and adjusted analysis of risk and protective factors for the prevalence of training in traditional, complementary, and integrative medicine. Santa Catarina, Brazil (N=386).

Variables	Categories (n)	W/ ICPH * training	p-value ^†^	Raw Prevalence Ratio (95%CI ^‡^)
**Sex**	Female (357)	132 (37.0%)	0.80	0.96 (0.73-1.27)
	Male (29)	10 (34.5%)	-	-
**Ethnicity** ^§^	Caucasian (346)	130 (37.6%)	0.55	0.92 (0.73-1.17)
	Asian-descendant. Mixed race or Afro-descendant (37)	12 (32.4%)	-	-
**Marital Status**	Married/ stable union/ cohabitate/ dating (265)	100 (37.7%)	0.57	0.95 (0.81-1.12)
	Separate/ Divorced/ Widowed/ Single (121)	42 (34.7%)	-	-
**Age group**	More than 61 y/o (10)	3 (30.0%)	0.002 ^||^	0.83 (0.74-0.94) ^¶^
	51 to 60 y/o (25)	10 (40.0%)		
	41 to 50 y/o (88)	40 (45.5%)		
	31 to 40 y/o (161)	61 (37.9%)		
	Up to 30 y/o (102)	28 (27.5%)		
**Time since graduation**	More than 60 months (278)	114 (41.0%)	0.005	0.80 (0.69-0.92)
	Less than 60 months (108)	28 (25.9%)		
Income (times the Minimum Wage)**	Up to 4 times MW(199)	68 (34.2%)	0.27	1.09 (0.93-1.27)
	More than 4 times MW(187)	74 (39.6%)	-	

*ICPH = Integrative Complementary Practices in Health; ^†^p-value = Chi-square test; ^‡^CI = Confidence interval; ^§^Three participants checked the option “none of the above”; ^||^Fisher’s exact test; ^¶^Breslow-Day test for interaction Ratio >0.05. The adjusted Prevalence Ratio was used; **Current minimum wage R$1,035.00, Brazil, 2021

Eighteen interviews and narratives were performed in the qualitative stage with the nurses implementing ICPH in the care provided to people with AH. According to the mixed-study sequencing, Figure 1 presents in a joint display the types of ICPH used for AH and the nurses’ reports concerning how they implement ICPH in their practices. 

**Figure 1 -:** Joint display of the types of traditional, complementary, and integrative medicine implemented among individuals with arterial hypertension. Santa Catarina, Brazil, 2021

	QUAN RESULTS*	QUAL RESULTS ^†^ (NARRATIVES)
ICPH ^‡^	n (%)	HOW ICPH ^‡^ ARE IMPLEMENTED
Auriculotherapy	40 (28.2)	*[...] some specific auriculotherapy points, such as kidney to drain that accumulated fluid,* shen men *, which gives you a feeling of wellbeing, a treatment for the problems one has, and which are not linked to high arterial pressure only* (CITRINE, 41 y/o, specialist, Grande Oeste). *I’ll do the anamnesis, see complaints, previous history of diseases, surgeries, and family history in the first appointment, and then, it’s when I also learn about hypertension. In auriculotherapy, I use that hypotension point and also take the chance to give orientation regarding health, diet, and exercises*(SELENITE, 40 y/o, MSc., Planalto Norte and Nordeste). *I’ve already used auriculotherapy and acupuncture with heart points and seen significant improvement* (AMETHYST, 42 y/o, specialist, Sul).
Acupuncture	12 (8.5)	*It also has an acupuncture point, which the patient can identify and manipulate* (AMAZONITE, 44 y/o, specialist, Grande Oeste).
Reiki	11 (7.7)	*In the nursing consultation, I don’t just explain about salt consumption, self-care, and exercises. I also treat with* Reiki *, massage, and auriculotherapy* (OBSIDIAN, 40 y/o, MSc. Grande Oeste).
Flower therapy	10 (7.0)	*[...] I use Bach remedies for hypertensive patients who experience difficulty sleeping and resting* (MOON STONE, 36 y/o, specialist, Planalto Norte and Nordeste).
Aromatherapy	9 (6.3)	*[...]lavender is hypotensive, verbena is sedative, and lemongrass, which we usually use as an individual inhaler, but they can be used in massage and compresses (WHITE QUARTZ, 36 y/o, specialist, Grande Florianópolis ).*
Phytotherapy	7 (4.9)	*[...] adjustments in diet and phytotherapy (SUNSTONE, 31 y/o, specialist, Grande Oeste).*
Meditation	7 (4.9)	*Hypertensive patients are often agitated, nervous, and worried, so the focus is to promote relaxation so that they decrease heartbeats, breathing, respiratory rate, and breathing pattern, which is often thoracic, and it also lowers blood pressure* (EYE OF TIGER, 39 y/o, MSc., Vale do Itajaí).
Moxibustion	5 (3.5)	
Cupping therapy	4 (2.8)	
Traditional Chinese Medicine	3 (2.1)	*[...] TCM [Traditional Chinese Medicine] guidelines, food, water use, teas, relaxation, and energy massage, massage on specific points, and I even teach them* Do in (ÁGATE, 50 y/o, MSc., Meio Oeste and Serra Catarinense).
Foot reflexology	3 (2.1)	*[...] through some manipulation points on the soles of the feet, there is a very important process of lowering blood pressure, reducing stress, edema, increasing diuresis, and improving the quality of medications (WHITE QUARTZ, 36 y/o, specialist, Grande Florianópolis).*
Anthroposophy	2 (1.4)	
Bioenergetics	2 (1.4)	
Chromotherapy	2 (1.4)	
Geotherapy	2 (1.4)	*Sometimes we use abdominal geotherapy to control high blood pressure (TOURMALINE, 46 y/o, Physician, Grande Florianópolis).*
Music therapy	2 (1.4)	
Yoga	2 (1.4)	
Ozone therapy	1 (0.7)	
Thermalism	1 (0.7)	

*QUAN = Quantitative; ^†^QUAL = Qualitative; ^‡^ICPH = Integrative Complementary Practices in Health

The nurses’ reports show that they sometimes use different ICPH practices or assess which one(s) to use, depending on each case. Therefore, many practices were mentioned and used differently as the primary intervention or to complement other practices.

**Figure 2 -:**
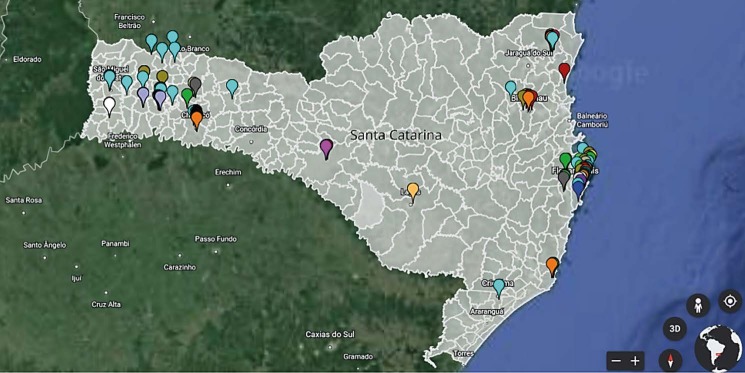
Geographic distribution of traditional, complementary, and integrative medicine adopted in the care provided to individuals with arterial hypertension. Santa Catarina, Brazil, 2021

Figure 2 shows how the ICPH included in the care provided to people with AH is geographically distributed in the state of SC, Brazil. 

ICPH is concentrated in the extreme west and east of the state of SC. Such concentration is possibly explained by the bias of the collaborators collecting data, as most live in cities in those locations.

Of the 142 nurses with ICPH training, 55 reported using these practices to care for people with AH. In order to integrate QUAN and QUAL data, a joint display was developed, including this specific portion of respondents and representative excerpts of their reports, focusing on the challenges and potentialities faced when implementing ICPH to manage AH Figure 3. 

**Figure 3 -:** Joint display presenting the challenges and potentialities experienced by nurses in the implementation of traditional, complementary, and integrative medicine in the care of people with arterial hypertension. Santa Catarina, Brazil, 2021

SUBJECT	QUAN RESULTS*	QUAL RESULTS ^†^ (NARRATIVES)	INTEGRATION
**CHALLENGES**		**The nurses seek training and pay for it themselves**	
	52 (94.5%) attended graduate studies	*I’ve sought most of my training myself because the city does not always make training programs available or even facilitate workers attending a training program* (SUNSTONE, 31 y/o, specialist, Grande Oeste).	Even though most nurses have a graduate degree or specialization, the initiative to seek training is personal, and managers provide little support.
		**Stigma due to a lack of knowledge**	
		*[...] professionals who do not know and undervalue ICPH* ^‡^ *, lack of knowledge, of reading and studying. We have to change our process because the health system will not be able to deal with how we currently treat people* (PYRATE, 35 y/o. MSc., Sul).	Even with proper training, the nurses must show their knowledge to defend the effectiveness of ICPH ^‡^ constantly.
		**Limited time to implement practices**	
	41 (74.5%) adopt ICPH ^‡^ in their daily practice, and most of these, 34 (61.8%) spend from one to four hours per week with ICPH ^‡^	*I work in an FHS [Family Health Strategy Unit], and implement ICPH* ^‡^ *in my practice less frequently than I’d like. It is implemented in a cross-sectional manner in my practice, it’s an additional tool. [...]* (EMERALD, 32 y/o, specialist, Grande Florianópolis).	Workload due to the many activities hinders the implementation of ICPH ^‡^ in daily practice when it is not a priority or is not seen as a cross-sectional tool to be used in the care and treatment of diseases.
		**Medicalization and biomedical culture**	
	23 (41.8%) nurses have a co-worker to discuss cases and determine the conducts related to treatments that include ICPH ^‡^	*[...] overcoming barriers that prevent the valorization of therapies. We have a society where pharmacological treatments are more powerful, especially economically. So, these treatments overlap with other issues. We are in a totally medicalized society; it’s more attractive to think that a pill will make you happy, healthy, and beautiful than recognizing that it all depends on ourselves, and our choices* (LÁPIS-LAZULI, 66 y/o, Physician, Grande Florianópolis). *The legal support nurses have when adopting the ICPH* ^‡^ *is seldom mentioned. We need to consolidate nursing as a profession to be appreciated as autonomous, independent, and trained professionals* (RUBY, 46 y/o, specialist, Grande Florianópolis).	The devaluation of ICPH ^‡^ on the part of managers and professionals may influence the number of professionals with ICPH ^‡^ training, contributing to the perpetuation of the biomedical care model. Additionally, there is a constant dispute between professions and the need to validate practices.
**POTENTIALITIES**		**Treatment adherence**	
	55 (100%) believe that having knowledge, contact with, or using ICPH ^‡^ positively influences the patients’ autonomy	*The benefit is better treatment adherence. It is easier to give up if you take only pharmacological drugs, so this support with ICPH* ^‡^ *is much more effective (MOON STONE, 36 y/o, specialist,* Planalto Norte and Nordeste).	ICPH ^‡^ contributes to treatment adherence, both pharmacological and non-pharmacological treatment, and also influences a decrease in the use of medications, encouraging patients to be more active in their self-care.
		**Patient autonomy and health promotion**	
		*ICPH* ^‡^ *helps a lot to change the [hypertensive patient’s] profile and quality of life. Many interventions are not implemented weekly; sometimes, they start weekly, but the idea is to generate autonomy and not replace the medication with integrative practices (TURMALINE, 46 y/o, Physician, Grande Florianópolis).* *[...] the benefit is one’s own wellbeing and quality of life (ONYX, 30 y/o, MSc., Grande Oeste).*	Changes in people’s lifestyles were observed when ICPH ^‡^ was implemented, besides an influence in decreasing risk factors such as stress, insomnia, and anxiety, for the development of other cardiovascular diseases.
		**Research**	
	44 (80.0%) performed studies on evidence-based practices to support a technique or practice; most 27 (4.1%) in the VHL ICPH ^§^	*It’s very good to be able to talk about and participate in studies addressing ICPH* ^‡^ *, because if we don’t talk about it, if we don’t show the population what we do, it won’t get disseminated (EYE OF TIGER, 39 y/o, MSc., Vale do Itajaí).*	The possibility of giving visibility to the practice and encouraging a reflection upon care practices and the incorporation of ICPH ^‡^.

*QUAN = Quantitative; ^†^QUAL = Qualitative; ^‡^ICPH = Integrative Complementary Practices in Health; ^§^VHL ICPH = Virtual Health Library on Traditional Complementary and Integrative Medicine

There are different responses regarding the adoption of ICPH in hypertensive crises. The nurses mentioned they had never used it due to a lack of knowledge, team adherence, management support, time, insecurity, or high demand. The reports reveal these aspects: *I have never heard about auriculotherapy to treat hypertensive crises, so I’ve never used it* (EMERALD, 32 y/o, specialist, Grande Florianópolis); *I’ve never used it in a hypertensive crisis, mainly because the team didn’t adhere. In these cases, the patient is immediately referred to a doctor and doesn’t return to me* (ROSE QUARTZ, 33 y/o, specialist, Grande Oeste); I *’ve never adopted ICPH to treat a hypertensive crisis. Not that I didn’t want to, but I’d ended up not doing it because the doctor would give a sublingual captopril. Then I’d consider going and making a small hole in his finger, bleed him, but then, what will the patient say?* (SODALITE, 45 y/o, specialist, Grande Oeste); *I’ve never attended a patient in hypertensive crises because I didn’t think I was apt to reach that point, I had little training time and felt unsafe. So I’d always go for medication. Also, I didn’t have much support from management, so I was insecure about anything happening and being misunderstood* (PIRITE, 35 y/o, MSc., Sul). Hence, the stigma nurses experienced for implementing ICPH and negative perceptions emerged in the reports. 

Among the positive experiences of implementing ICPH to care for hypertensive crises, auriculotherapy bleeding stands out: *Hypertensive patients are usually very yang and agitated. Then you bleed the ear, and in a few minutes, the pressure drops* (AMAZONITE, 44 y/o, specialist, Grande Oeste); *I’ve seen hypertensive patients having a hypertensive crisis and used auriculotherapy, a bleeding technique. We have some very important hypotensive points, and we get a very significant decrease in blood pressure. But when the crisis is linked to anxiety, rather than only physiological processes, we also use medication* (WHITE QUARTZ, 36 y/o, specialist, Grande Florianópolis). Acupuncture, bioenergetics, *Reiki*, flower remedies, and Geotherapy were also mentioned in the nurses’ practice when caring for hypertensive crises: *I’ve already used bioenergetics,* Reiki *, and flower remedies for hypertensive crises, we have a specific emergency flower remedy, and it provides support for the person to determine the point to which she will go, sometimes at the hospital or at the health unit* (MOON STONE, 36 y/o, specialist, Planalto Norte and Nordeste); [...] *bleeding with the needle according to the Chinese medicine practice. I assess where this process is somatizing, we adapt according to where he experiences more discomfort, and we use geotherapy a lot such as cephalo sacral poultice* (TURMALINE, 46 y/o, Physician, Grande Florianópolis); [...] *bleeding at specific acupuncture points. I check the place where I need to do my intervention according to the five elements. I use VG14 or R1 a lot, which is a point that decreases the energy concentrated at the top. Fingertip points show fantastic results in decreasing blood pressure* (AGATE, 50 y/o, MSc., Meio Oeste and Serra Catarinense). 

Nurses use health education to encourage self-care and co-responsibility. Changes in lifestyle, such as increased fluid intake and dietary care, were associated with the use of ICPH, promoting health, avoiding hypertensive conditions, and preventing injuries caused by AH and its complications.

Additionally, one should consider the support provided by ICPH Integrative Complementary Practices in Health when associated with care practices, which is something positive, with the potential for nurses to decrease the number of allopathic medications, adjust the doses of antihypertensive medications better, and strengthen the bonds between users and the health service, promoting adherence to non-drug treatments, such as modifying lifestyle.

## Discussion

The profile of nurses with ICPH Integrative Complementary Practices in Health training in Santa Catarina, Brazil, is similar to that shown by the Nursing Profile Survey in Brazil ^( 14)^ . As for the participants’ gender, the predominance of women reflects an age-old aspect of identifying the practice of care as being of a female nature rather than a profession. Historically and culturally, nursing is a field composed of women who used to provide care as a charitable or religious activity, or from a layman’s perspective, consistent with low remuneration ^( 15)^ ; such identity aspects affect the profession even today. 

Discussions concerning ICPH in Brazil have occurred since the 1970s, but only in 2006 was the National Policy on Integrative and Complementary Practices ( *PNPIC* in Portuguese) instituted ^( 16)^ . The recent implementation of the PNPIC may explain why older nurses are less likely to seek ICPH training than younger nurses, suggesting that public policies influence the search for training in different fields. 

The nursing field addresses all stages of life and is present in all Brazilian cities. Nurses represent more than half of all health professionals and are essential for delivering quality health care ^( 17)^ . ICPH is an important tool to meet the principle of integrality and can potentially expand access and supply, besides the qualification of health services. The nurses’ reports regarding how ICPH is incorporated into the care provided to individuals with AH reveal a concern with looking at patients from an integral and comprehensive perspective to promote health, considering that “[...] the ICPH’s central focus is based on human responses, rather than diseases” ^( 5)^ . 

Because AH is a modifiable condition and a public problem at a global level, investing in strategies to prevent diseases and recover health can improve quality of life. A recent model presents strategies for the 80-80-80 goal (80% of individuals with AH are screened and become aware of their diagnoses; 80% of these are prescribed treatment; and 80% of those receiving treatment achieve BP control targets), which is intended to decrease all-cause mortality from 4% to 7% (i.e., 76 to 130 million deaths are expected to be averted between 2022 and 2050). Most risk factors for AH are modifiable, and the target is directly related to achieving global targets to decrease overweight and obesity, the harmful use of alcohol, and high salt consumption ^( 18)^ . The possibilities to achieve such targets include using ICPH ^( 3)^ . 

Health promotion to decrease risk factors is an important global and national target for reducing NCDs. Considering primary prevention, stress control is a non-drug treatment recommended for people with AH ^( 2)^ . Psychological factors deserve attention. A recent study reports that 60.6% of women feel stressed, and 33.6% presented AH ^( 19)^ . These aspects reflect the reports of nurses adopting ICPH to deal with emotional issues, such as sleep and rest, stress, depression, and anxiety, which boost BP alteration-related imbalances. 

Auriculotherapy stood out among ICPH practices adopted in the care provided to people with AH. Auriculotherapy is an acupuncture microsystem in which needles, crystals, seeds, laser, Moxibustion, or infrared are used in ear points to treat the signs and symptoms of varied diseases ^( 20)^ . 

Regarding the procedures mostly frequently performed, data concerning ICPH implemented in Brazilian Primary Health Care in 2019 ^( 21)^ show that auriculotherapy was the procedure most frequently applied in a universe of 628,239 procedures, with 423,774 sessions, followed by 129,207 sessions of acupuncture with insertion of needles, corroborating this study’s findings. 

Research addressing the effectiveness of ICPH has shown positive results ^( 22- 24)^ , among which is auriculotherapy, in which some protocols guide the practice in the care provided to people with AH ^( 25)^ ; kidney, hypotensive, *shen men*, and heart are some auricular points addressed by the nurses. 

One of the auricular acupuncture techniques, known as bloodletting, is performed by piercing the ear capillaries with a needle to release a few drops of blood ^( 25)^ . Bloodletting at the apex of the ear is the most frequent practice to treat hypertensive crises. A study conducted in Santa Catarina used bloodletting only once in the brain reflex point and showed a reduction in systolic BP in 80% of the volunteers ^( 26)^ . 

Evidence shows the positive effects of auriculotherapy on AH ^( 4- 5)^ . However, studies with a low methodological quality indicate the need for more investments, considering that many potentialities are pointed out in clinical practice. In addition, given the specificity of the ICPH paradigm, traditional research methods may be challenging to apply. 

“Even though studies addressing ICPH are still scarce in Brazil, research has grown in recent decades. Such growth is evidenced by analyzing three aspects: the promotion of research, research groups/lines, and publications” ^( 27)^ . This reflection is relevant, considering that only in 2013, ICPH research in Brazil had its first public notice for specific funding ^( 28)^ . 

Research has been growing exponentially, and broad international studies have been developed in the nursing field, such as the Erasmus+ program, which investigates integrative nursing to improve training in the field ^( 29)^ . Research is an important form of dissemination and promotes the growth of ICPH, which is widely used and researched in developed countries. ICPH has been provided within SUS free of charge in Brazil since the *PNPIC* ^( 16)^ was implemented, though some barriers impede its consolidation. 

The nurses’ reports repeatedly indicated a lack of management support. A study addressing the coordinators of 45 basic health units shows that a lack of knowledge about ICPH is often observed despite it being supplied within the public health system. Note that the concept of biomedical care prevails even in units where ICPH is supplied, showing that the fact that ICPH is available in the services by itself does not change care practices ^( 30)^ . 

Few managers recognize the supply of ICPH, reinforcing the professionals’ protagonism, as they are responsible for the expansion and supply of ICPH within SUS ^( 31)^ . This fact possibly explains the supply reported in the ICPH National Monitoring Report in the Health Information Systems ^( 26)^ , corroborated by the participants’ reports. 

Such aspects concern another challenge the nurses reported concerning a lack of support for them to obtain ICPH qualification and training, in line with a national survey showing that professionals self-finance their ICPH training ^( 31)^ . 

Another factor the nurses reported that aggravates the many challenges already faced when incorporating ICPH into the care of people with AH is associated with prejudice and stigma. Note that millenary medicine and care practices lost room with the advancement of modern medicine, boosting the pharmaceutical industry and the use of medications. The advancements and ICPH are paramount in health care; however, the current indiscriminate medicalization and the notion that pharmacological treatment is the only resource available, disregarding the possibility of associating non-drug treatments, have been challenged.

Considering the possibilities of care, prevention, and healing with “other medicines,” a review assessed the adoption of ICPH to care for chronic diseases, indicating an inclination towards its use in managing AH and diabetes mellitus and more frequent use of phytotherapy. However, although the legislation provides for various practices, ICPH is still seldom adopted, and the lack of professional training is apparent ^( 32)^ . 

The nurses’ reports show a lack of knowledge concerning legal support, which is of concern, considering that the nursing field pioneered recognizing ICPH as a professional practice ^( 7)^ . The dispute between professions is commonplace, and some ICPH practices are still in an accreditation process that requires advancements in the profession’s legal aspects. However, several practices are among the specialties recognized in COFEN Resolution No. 625/2020(8). In addition to the COFEN/COREN system, the Brazilian Association of Acupuncturist Nurses and Integrative Practice Nurses (ABENAH) deserve mention as these are working toward this direction. 

The potential highlighted in the nurses’ reports concerns a decrease in medication use, also reported in another study ^( 33)^ . However, this quality of ICPH cannot be generalized, as it has multiple dimensions. Some of the elements that facilitate a decrease in medications are configured in a “[...] greater tendency of horizontal clinical relationships, encouraging patients to participate, with an expanded and holistic approach to problems, addressing spiritual aspects, and appreciating the individuals’ stories and experiences, as this indirectly facilitates a reflection and understanding of life contexts and singular experiences, potentially supporting a decrease in the number of medications” ^( 34)^ . 

This aspect is in line with autonomy, another potentiality highlighted, and ICPH can positively influence one’s ability to make decisions regarding care and how to meet health needs ^( 35)^ . Meanwhile, a review concluded, “[...] professionals and patients look to the ICPH possibilities to improve their health and quality of life. In this sense, the dissatisfaction of many users with the biomedical model may increase interest in ICPH as a support for health care. Furthermore, the users’ autonomy to choose complementary treatments makes them feel protagonists and co-responsible for their care” ^( 36)^ . 

When providing and performing ICPH, the focus must be on the integral care provided to people. However, in terms of research, narrowing down to certain diseases, as we did in this study, may help advance knowledge. A gap and possibility for future investigations is the development and implementation of nursing protocols based on the various ICPH practices to lead to care practices for treating AH.

The main limitation of this study concerns the restricted location from where the nurses were recruited, considering different experiences may be found in other regions of Santa Catarina, Brazil, and would allow a deeper understanding of the context addressed here. Nevertheless, nurses from six of the seven health macro-regions were interviewed, and the minimum sample needed to ensure statistical significance was obtained.

This study has implications for advancing scientific knowledge in healthcare and nursing, as it shows the previously unknown profile of nurses with ICPH training in Santa Catarina, Brazil. It also contributes to the understanding of the ICPH incorporated in the care provided to people with AH and its potential, contributing to evidence already available, strengthening the role of nurses in the implementation of ICPH, considering that the nursing field is a pioneer in the implementation of ICPH and training according to its principles.

## Conclusion

This study enabled identifying and understanding what ICPH is and how nurses implement it in the care provided to people with AH in Santa Catarina, Brazil.

Among the 386 participant nurses, 14.2% reported incorporating ICPH into the care of people with AH, with auriculotherapy being the practice most frequently implemented, followed by acupuncture and *Reiki*, to establish integral balance, with effects on anxiety, stress, sleep, rest, and influencing a decrease in blood pressure. Therefore, various practices were reported and implemented differently, whether ICPH was the primary intervention or complemented other care practices. 

The nurses’ concern with lifestyle stood out in their reports. It was a variable considered in care planning, and health education was used to encourage self-care and co-responsibility. ICPH was mentioned as a preventive measure to prevent AH or its worsening. The auriculotherapy bleeding technique with a hypotensive effect stood out in the care provided to hypertensive crises.

Among the challenges experienced by nurses when applying ICPH in the care provided to people with AH, the biomedical culture, centered on disease and medicalization, was frequently mentioned, and nurses emphasized that health professionals, managers, and the general population undervalued ICPH. Additionally, the reports indicated a deficit in training on the subject.

Research is considered one of the potentialities because it has the potential to provide further visibility to this practice and encourages a reflection upon care practices that include ICPH. The implementation of ICPH can contribute to reducing the use of allopathic medications, promoting greater autonomy, improving quality of life, and reducing risk factors for other cardiovascular diseases.
